# Mechanical Performance of 3D-Printed Biocompatible Polycarbonate for Biomechanical Applications

**DOI:** 10.3390/polym13213669

**Published:** 2021-10-25

**Authors:** Giovanni Gómez-Gras, Manuel D. Abad, Marco A. Pérez

**Affiliations:** IQS School of Engineering, University Ramon Llull, Via Augusta 390, 08017 Barcelona, Spain; giovanni.gomez@iqs.url.edu (G.G.-G.); manuel.abad@iqs.url.edu (M.D.A.)

**Keywords:** additive manufacturing, biocompatible polycarbonate, material characterisation, mechanical properties, fatigue

## Abstract

Additive manufacturing has experienced remarkable growth in recent years due to the customisation, precision, and cost savings compared to conventional manufacturing techniques. In parallel, materials with great potential have been developed, such as PC-ISO polycarbonate, which has biocompatibility certifications for use in the biomedical industry. However, many of these synthetic materials are not capable of meeting the mechanical stresses to which the biological structure of the human body is naturally subjected. In this study, an exhaustive characterisation of the PC-ISO was carried out, including an investigation on the influence of the printing parameters by fused filament fabrication on its mechanical behaviour. It was found that the effect of the combination of the printing parameters does not have a notable impact on the mass, cost, and manufacturing time of the specimens; however, it is relevant when determining the tensile, bending, shear, impact, and fatigue strengths. The best combinations for its application in biomechanics are proposed, and the need to combine PC-ISO with other materials to achieve the necessary strengths for functioning as a bone scaffold is demonstrated.

## 1. Introduction

In parallel with the development of biocompatible materials, the technological evolution of additive manufacturing (AM) has made possible a beneficial approach between both fields, which has opened new horizons for applications related to biomechanics and biomedical engineering [1,2]. Furthermore, this technological advance and transformations have attracted the attention of the health sector to AM, especially in those areas in which a high degree of personalisation of treatments and devices is decisive for its success. This is the case, for example, of orthopaedics and rehabilitation, where AM has been advancing in recent years [3,4].

The immediate repair of bone disorders has been an ancestral clinical need, which has required the use of considerable resources in the field of medicine. Although many and various solutions have been successfully implemented, both internal and external treatment of bone defects remains a scientific challenge, as materials with adequate mechanical performance and favourable biological properties are required simultaneously [5]. However, with the current development of engineering-grade polymeric materials and the possibility of developing custom components by AM, new and versatile applications have been revealed for the biomechanical field in general, and orthopaedic in particular [6].

In addition, the development of the health sector, which has significantly increased life expectancy, has led to an increase in the elderly population, which is estimated to be 90% with bone problems after 40 years [7]. For example, from an economic perspective, musculoskeletal disorders totalled around EUR 228 billion in treatments, interventions, and research in 2008 [7].

AM currently allows the artificial geometrical reproduction of bones, exoskeletons, or anatomically identical parts to be replaced or reinforced. Several studies have approached this perspective, providing interesting analyses about the multiple applications of 3D printing with polymers of different nature for anatomical models or tissue engineering [8], in the use of metal alloys for orthopaedics and dentistry [9], in the control of necessary stiffness and porosity to manufacture bone implants with functional success [10,11], as well as the management of the entire range of AM technologies, indicating the most suitable application areas [12,13]. However, obtaining AM materials not rejected by the body is not a trivial task.

The bone can be considered a material composed of hydroxyapatite and type I collagen [14], showing inherent anisotropy and heterogeneity, which makes it difficult to establish a generalised value of the modulus of elasticity since it can vary between 2 and 30 GPa, depending on the type of bone, its porosity, and its direction [15]. Thus, despite the effort to understand the architecture of biological bone [16], the design of bone inserts, and their use in external immobilisation for disorders requiring intensive rehabilitation, there are still many relevant limitations that slow down the widespread insertion of these new materials in applied medicine.

New expectations have been created as AM technologies are being consolidated. AM generally consists of layer-by-layer deposition of material in a controlled manner until a three-dimensional structure of high geometric complexity is formed. Characteristics such as slenderness, internal casting, changeling, variable thicknesses, irregular shapes, and the reproduction of nature (search for ergonomics, aerodynamics, hydrodynamics, etc.) are challenges that conventional manufacturing methods (subtractive and conformative) have not addressed with the same success [17,18]. In addition, the expected customisation in the design of prostheses or orthoses for their complete adaptation does not make this process more costly, which makes it ideal for this sector, in which end products with high added value are desirable [19]. These benefits have facilitated investment in preclinical testing and clinical applications, as well as new perspectives for bone implants based on AM [20].

Nowadays, it has been shown that the imitation of the structural characteristics of human biological bone using substitute orthopaedic polymers has advantages for its implantation [5]. The anatomical complexity, details of the support structure, and variations in the densities, among other peculiarities, are characteristics that can be solved with the reproduction of a model by AM. While reducing the associated cost compared with the conventional manufacturing process, this total customisation makes it possible to obtain a prosthesis or an orthosis equal to the defect to be supplanted or corrected. This advantage undoubtedly contributes to its successful adaptation.

In orthosis design, the use of customised models is much closer to reality, given the lack of risk involved in their implantation. However, in the case of bone insertions with artificial materials, the applicability is not direct. The strategies commonly used for replacing bone defects are allografts and autografts due to the highest osteoconductivity and osteoinductivity [21]. Autografts are bone transplants of the patient’s bone, from one area of the bone skeleton to another. In contrast, in the allograft, the donor is of the same species but genetically different. However, several associated disadvantages include limited bone supply, donor site morbidity, or possible transmission of bacterial diseases leading to rejection [22]. 

Part of these drawbacks could be addressed with artificial AM inserts that, in addition, allow the manufacture of complex microstructures imitating, e.g., the natural porosity of the bone, necessary for cell proliferation that must lead to the regeneration of the affected area. Studies with widely used materials such as PLA [17,23] show that scaffolds designed for these applications, with a porosity of around 30–50%, offer successful cell proliferation and osteoconduction results. However, the mechanical performance cannot be equated to those of bone tissue. In other words, the concept of meta-biomaterial [24], taking advantage of the auxetic characteristic of the proposed configuration, is only addressed to tackle problems of stiffness–expansion in the bone–implant contact or to address the mechanical performance under conditions of quasi-static and cyclic loads [25]. Nevertheless, in studies on polymers, most authors conclude that thermoplastics could be useful if they are mixed with other materials that help them achieve the required strengths [18,21].

The biocompatible PC-ISO polycarbonate is currently a material postulated as a promising candidate for part of these applications. It encompasses a series of outstanding properties for use in the health industry, especially those in direct contact with humans, due to their compliance with ISO 10993 [26] and USP Class VI certifications (Class Testing standards by the United States Pharmacopeia and National Formulary). This polymeric material in filament form can be used for printing by FFF [23,27], one of the most versatile AM technologies [22]. However, to precisely define its application areas, it is necessary to understand its mechanical performance thoroughly. It has already been shown in previous studies with engineering-grade polymers [28,29,30] that the variability of its properties is highly dependent on printing conditions. It may even be essential to use a solvent to eliminate the support material necessary to print highly complex geometric structures such as those proposed for this type of application, without this implying a deterioration of the mechanical behaviour [31,32].

Accordingly, this work aimed to investigate the mechanical performance of PC-ISO 3D-printable synthetic polymer as a potentially competent structural material for use in applied biomechanics. A detailed examination was made of the parametric configuration to complete this objective, defining the combination of parameters that provide optimal mechanical performance beyond the general data reported on manufacturer datasheets [33]. This information determined the extent to which this material can meet the structural requirements and mechanical stresses expected in such applications and, in turn, clarified the magnitude of the contribution that other materials combined with PC-ISO would have to make to meet user expectations. Therefore, a comprehensive mechanical characterisation of PC-ISO is presented throughout the study, including analysis into the effect of FFF printing parameters on its static, dynamic, and fatigue performance. This allows a complete understanding of its limitations and strengths and provides the scientific community with essential information to determine in which bio-structural applications this biocompatible material would be most appropriate.

## 2. Materials and Methods

### 2.1. Biocompatible Polycarbonate

Polycarbonate (PC) is a thermoplastic polymer with a chemical structure of bisphenol-A molecules linked to carbonate groups in a molecular chain. Biocompatible polycarbonate PC-ISO (polycarbonate-ISO) is an engineering-grade polymer used to manufacture food and drug packaging components and medical devices. The material can be sterilised with gamma radiation and ethylene oxide and is especially attractive because it meets ISO 10993 and USP Class VI standards used to assess biocompatibility. Particularly, ISO 10993 tests how a material interacts with blood and body fluids, while USP Class VI tests materials by implanting them into subjects and monitoring any signs of reactivity [26].

### 2.2. Test Specimens

**Figure 1:**
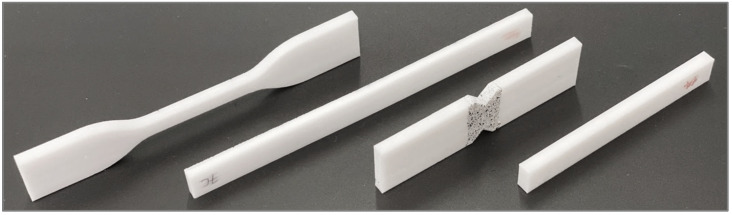
Test specimens’ designs, with dimensions according to the corresponding ASTM standards for each characterisation test. From left to right: tensile testing (ASTM D638), flexural testing (ASTM D790), shear testing (ASTM D5379), fatigue and impact testing (ASTM D7774 and ISO 179-1).

The test specimens were designed (Figure 1) according to ASTM or ISO standard specifications. The specimens’ dimensions of each test configuration are listed in Table 1. 

### 2.3. Experimental Design and Specimen Manufacturing

**Figure 2:**
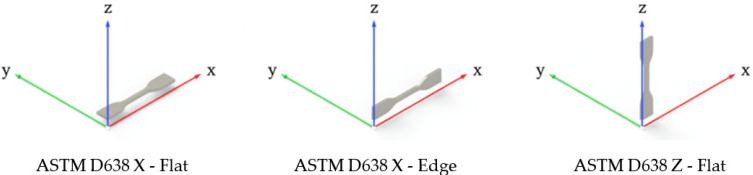
Representation of the printing sample orientation combinations for the ASTM tensile test.

**Figure 3:**
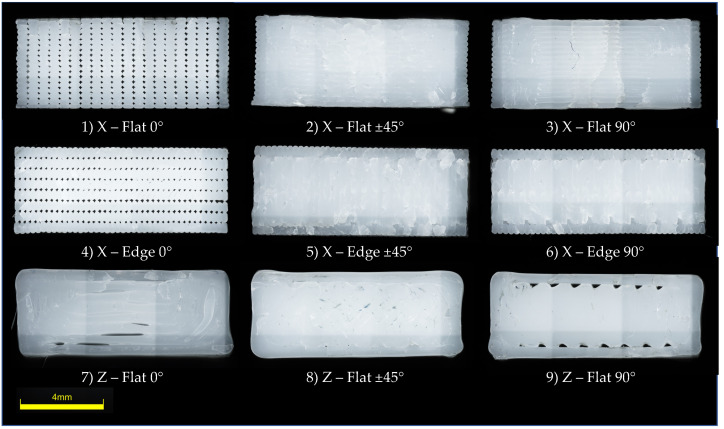
Transversal cross sections of X-Flat, X-Edge, and Z-Flat samples. See Figure 2 for orientation details.

Tensile, shear, flexural, impact, and fatigue testing were addressed to examine FFF building parameters’ role in the mechanical performance of PC-ISO FFF samples. The building parameters chosen for this study were sample orientation, printing orientation, and raster angle. Other parameters such as contour rasters (1 contour), raster-to-raster air gap (0 mm), and surface style (normal) were kept constant. A slice height of 0.254 mm (0.010 in) corresponding to a T16 tip was used. The chosen printing parameters have been made based on a previous work [28], in which it was proved that were no notable differences between X and Y printing orientations. Hence, orientation Y was excluded. Z-Flat and Z-Edge configurations correspond to equivalent test samples since the layers are printed on the same plane (manufacturing plane). Accordingly, only Z-Flat (ZX) configurations were studied. Five samples were printed for each configuration. Three specimens and four loads are used per configuration for flexural fatigue tests, as specified on the corresponding standard. This led to a total amount of 322 tested samples. The design of experiments is shown in Table 2, and the manufactured part orientations for ASTM D638 tensile test are represented in Figure 2 for a better understanding. In Figure 3, transversal cross sections of X-Flat, X-Edge, and Z-Flat samples are depicted. 

Samples were fabricated using Stratasys Fortus 400 mc FDM equipment. This printer is equipped with a temperature chamber that ensures a controlled temperature during the entire manufacturing process. This controlled environment is crucial, as it significantly enhances the interlayer cohesion between adjacent building layers. Regarding the supplier indications, the optimum working conditions for postprocessing PC-ISO require an oven temperature of 145 °C. The extrusion temperature for the model material (PC-ISO) is 365 °C. Once the specimens were printed, support structures were removed, and the mass and dimensions of each sample were measured before testing.

### 2.4. Experimental Testing

**Figure 4:**
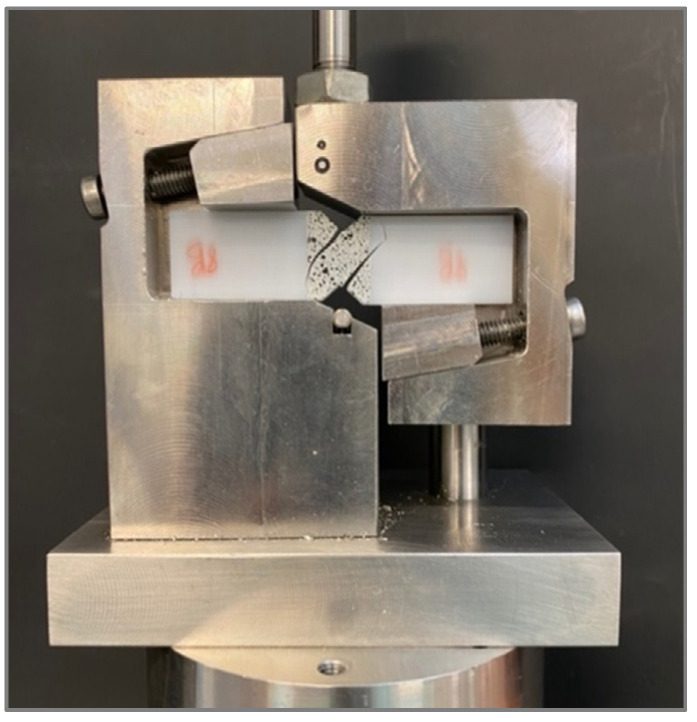
Experimental setup for ASTM D5379 shear test standard with digital image correlation equipment.

Tensile, shear, and bending tests were performed using Zwick 30 kN equipment (ZwickRoell, Ulm, Germany). Tensile tests were conducted following the ASTM D638 standard [34]. Specimen type IV was chosen with a thickness of 4 mm. The yield point was determined with an offset method of 0.1% strain. The results of tensile modulus, yield stress, yield strain, tensile strength, and strain at tensile strength were reported. For shear testing, ASTM D5379 test standard [35] was followed (see Figure 4).

A 3D digital image correlation with two GigE MAKO G-507B digital cameras with APO-Xenoplan 1.4/23–0903 lens was used to measure full-field shear strain. Specimens were previously sprayed with a black-and-white stochastic pattern. The system was calibrated with a GOM Correlate CP20/MV55x44 panel. The video sequences were treated with GOM Correlate Professional software to analyse the full-field strain of the samples. For the shear test, the yield point was estimated using the offset method with a strain of 0.2%. Shear modulus, yield point data, shear strength, and strain at shear strength values were reported. 

**Figure 5:**
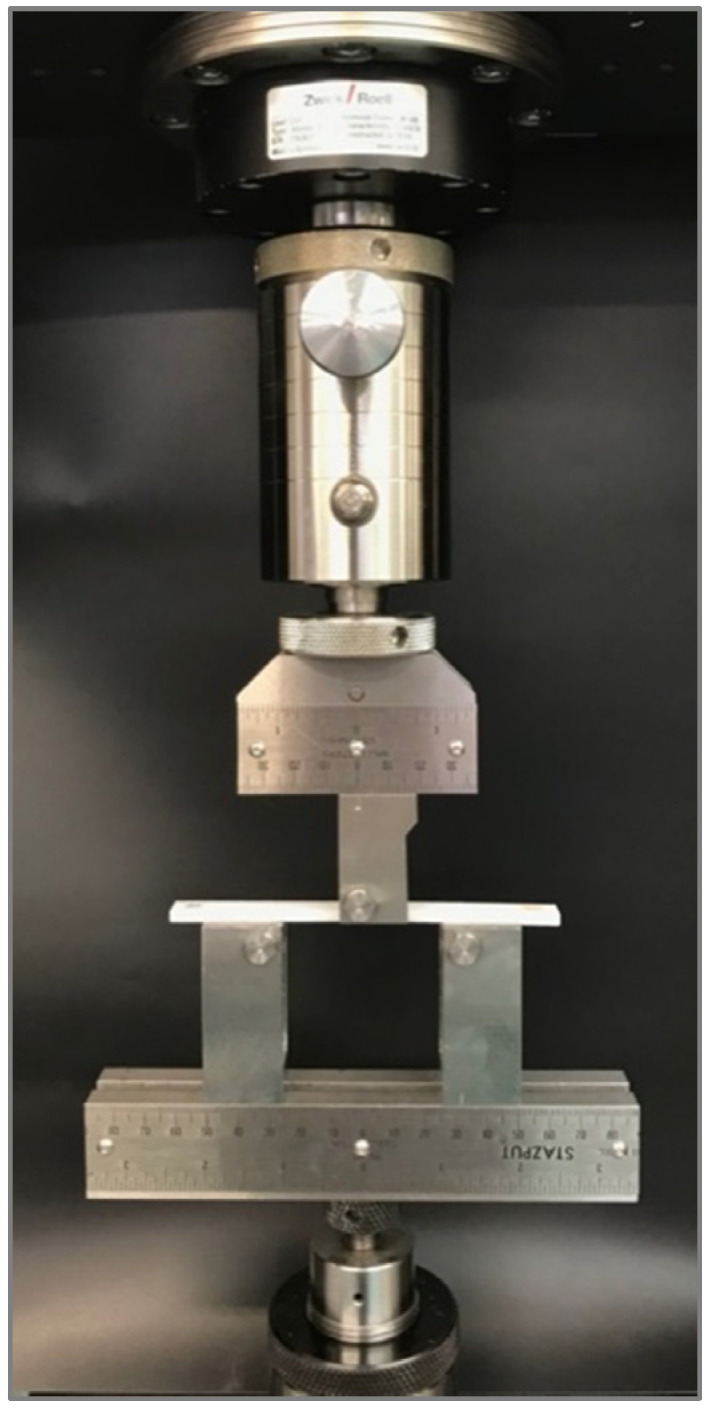
Experimental setup for ASTM D790 three-point flexural testing.

For three-point flexural testing (see Figure 5), the ASTM D790 test standard [35] was followed with a crosshead displacement rate of 1.71 mm/min. The yield point was determined with an offset method of 0.1% strain. Flexural modulus, yield stress, yield strain, flexural strength, and strain at flexural strength results were reported. 

Charpy impact testing was conducted using ZwickRoell test equipment (ZwickRoell, Ulm, Germany) with a 5 J pendulum. The testing procedure followed the ISO 179-1 standard [37]. Unnotched samples were fabricated; however, before testing, specimens were V-shaped notches. Since dimensioning of the notch and the accuracy of its positioning influence the measured energy, a ZwickRoell automatic notch-cutting machine was used to accurately produce the V-shaped notch on one side of the specimen, in accordance with the ISO standard. Measures of fracture energy were collected after tests. 

**Figure 6:**
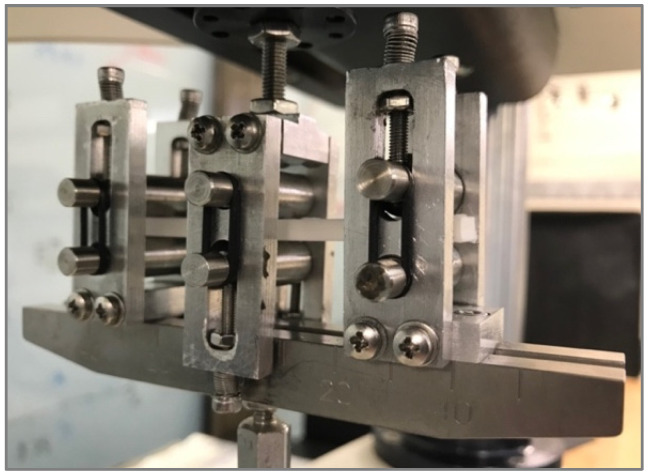
Experimental setup for ASTM D7774 three-point fatigue testing.

Lastly, three-point fatigue tests were conducted in a BOSE Electroforce 3200 dynamic equipment (Bose Corporation, Framingham, United States), following the ASTM D7774 [38] (see Figure 6). The fatigue-bending fixture followed procedure A, as defined in the ASTM D7774 standard, consisting of two double-sided supports and a double-sided loading nose with a 5 mm radius, equivalent to that of the ASTM D790. Fatigue samples were tested for four loading conditions, corresponding to 80%, 60%, 40%, and 20% of the flexural strength of each corresponding configuration. Life cycles of each test were reported. 

In addition, surface hardness tests were conducted following the ASTM D785 [39], obtaining an average value of the surface hardness Rockwell R 114 ± 4. The standard deviation of this test is considerable since the accuracy of the test is highly dependent on the indentation location (on the filament or between filaments).

## 3. Results and Discussion

### 3.1. Tensile Mechanical Performance

Table 3 shows the details of mass and printing time for each tensile test configuration. As seen, the higher manufacturing times correspond to the upright samples printed in Z (configurations 7, 8, and 9), as expected, since these are formed by a much higher number of layers than those printed in the other axes, and because, between layers manufacturing, purging of the tips occurs. Moreover, the standard deviations are low so that the accuracy of the print is guaranteed. Table 3 also lists the tensile modulus, maximum stress, and strain at maximum stress for each test configuration. Results correspond to the arithmetic mean of five specimens with the same combination of parameters.

**Figure 7:**
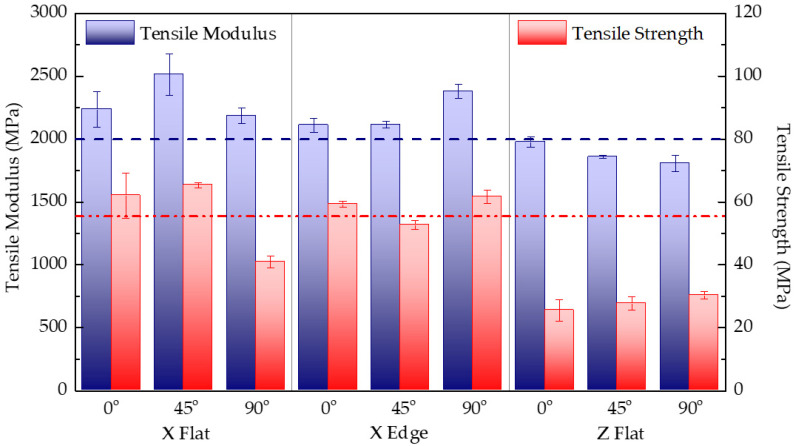
Comparison of the tensile modulus and tensile strength for each tested print configuration. Red and blue dashed lines stand for PC-ISO datasheet [33] reported tensile modulus (2000 MPa) and tensile strength (57 MPa), respectively.

Figure 7 shows the experimental results of tensile modulus and tensile strength for each tested configuration, together with the reference data reported by the manufacturer for PC-ISO [33]. As seen, tensile moduli are close to the reference modulus (2000 MPa) regardless of the test configuration. Overall, tensile results reveal an orthotropic stiffness behaviour, but it does not become as outstanding as initially expected. This fact is attributed to the quality of the joints between coplanar filaments (intralayer unions) and adjacent layers (interlayer unions) due to the use of a temperature chamber that allows reducing the thermal shock that occurs when the extruded filament is deposited and contacts the previously built layer. It should be noted that, although the amount of material used in each configuration is similar, there are notable differences in manufacturing times. The Z configuration presents the worst mechanical tensile performance and demands the longest time to print.

However, significant differences are found in the strength data analysis, particularly for Z-printed samples whose strength values are clearly below the reported reference strength (57 MPa) [33], thus showing the weakness of the upright printing configuration. Furthermore, the analysis of the strain data depicted in Table 3 also displays an important disparity between configurations 1 through 6, where layers are parallel to the load direction, and configurations 7–9, where layers are perpendicular to the load direction. As observed, the strain data for the Z samples deviate considerably from the value reported by the manufacturer (4%). This result is because the fracture in the Z-direction tests leads to the separation of two adjacent layers, resulting in a brittle type of failure. Hence, results state the lower resistance of the joints between layers compared with that of the filament polymer itself.

### 3.2. Flexural Mechanical Performance

**Figure 8:**
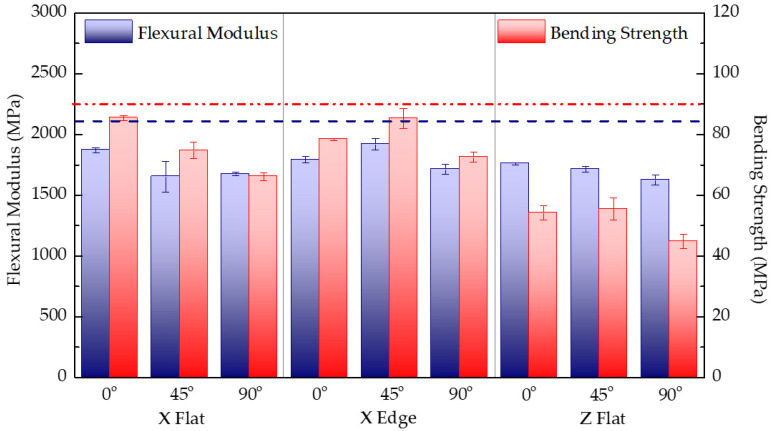
Comparison of the flexural modulus and bending strength for each tested print configuration. Red and blue dashed lines stand for PC-ISO datasheet [33] reported flexural modulus (2100 MPa) and bending strength (90 MPa), respectively.

Table 4 outlines the mass and printing time results, together with the flexural modulus, maximum stress, and strain at maximum stress for each test configuration. Results correspond to the arithmetic mean of five specimens with the same combination of parameters. Figure 8 shows the experimental results of flexural modulus and flexural strength for each tested configuration, together with the reference data reported by the manufacturer for PC-ISO. Overall, no clear evidence of printing parameters such as specimen orientation or raster direction can be highlighted from the results obtained. Regarding stiffness data, results show no outstanding variation between samples print configuration, but all results are below the reference modulus (2100 MPa) reported on the PC-ISO datasheet [33]. The same occurs with strength results, whose values are below 90 MPa reported flexural strength. Again, significant differences are found in the strength of Z-printed samples in which the fracture surface occurs between layers. A similar conclusion can be drawn regarding strain data since strain values at maximum stress values of Z-printed samples are significantly lower than X samples, as is the case with the tensile performance.

For X-Flat samples, the lower strength performance is observed for 90° configurations since samples have intralayer filaments parallel to the stress plane. In contrast, the 0° configuration specimens have the infill rasters perpendicular to the stress plane. Hence, the results indicate that solid samples’ resilience and bending tenacity are lower when the intralayer unions support the stress, as expected. Thus, the maximum resilience is generally achieved when the orientation of the filaments matches with the direction of the tensioned fibber (raster angle of 0°).

### 3.3. Shear Mechanical Performance

**Figure 9:**
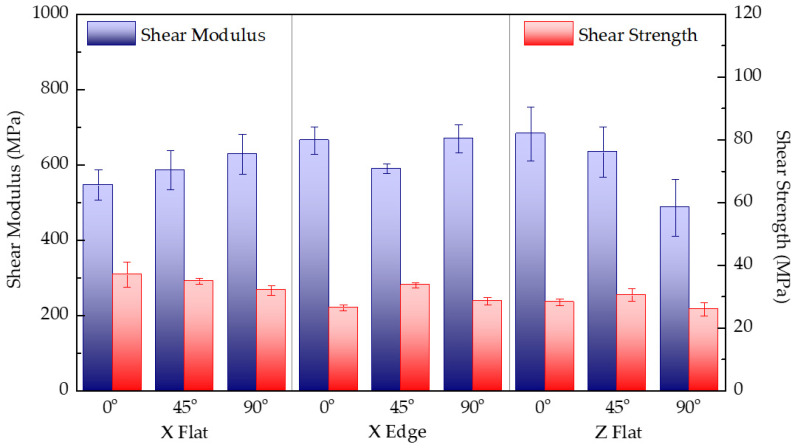
Comparison of the in-plane shear modulus and shear strength for each tested print configuration.

Table 5 lists the mass and printing time results, together with the shear modulus and maximum stress of each test configuration. Results correspond to the arithmetic mean of five specimens with the same combination of parameters. Figure 9 shows the experimental results of the in-plane shear modulus and shear strength of each tested configuration. For this test condition, there is no reference value reported by the manufacturer.

Differences in shear stiffness and strength do not show a clear trend, despite the different infill filaments arranged and different alignments due to the printing parameters of each configuration. Differences are attributed to the variation in the effective cross section of samples due to the arrangement of filaments and layers. Figure 4 shows the detail of the cracking of a sample during the shear test, coincident with the principal stresses caused in the shear loading state. As the infill filaments are deposited perpendicularly to the resistant cross section, planes slide and shear during the test until the intralayer unions or the interlayer ones fail. In upright samples, the superior strength of this solid configuration is reached when the number of intralayer filament unions in the effective cross section is higher. Lastly, as shown, the shear strength is significantly lower than tensile and bending strengths.

### 3.4. Impact Mechanical Performance

**Figure 10:**
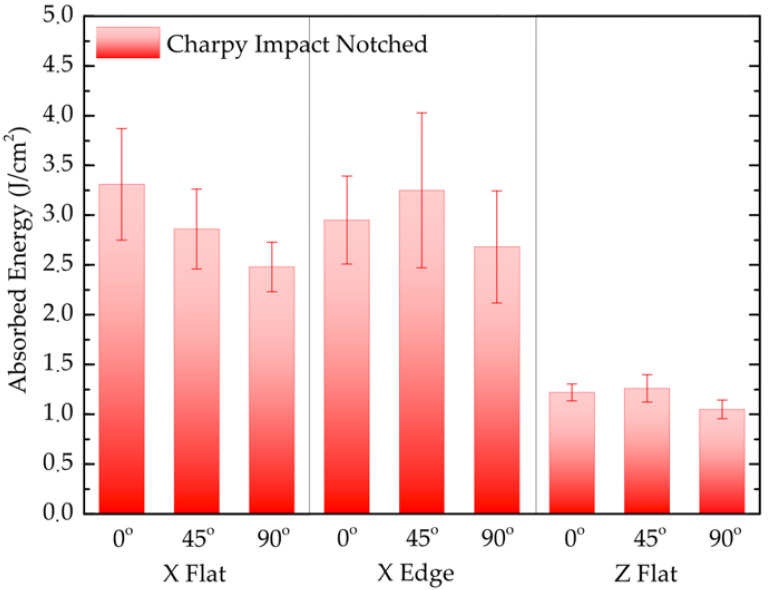
Comparison of the Charpy absorbed energy for each tested print configuration.

Table 6 lists the mass, printing time, and Charpy impact results for each test configuration. Results correspond to the arithmetic mean of five notched specimens with the same combination of parameters. Figure 10 shows the experimental results of Charpy impact absorbed energy for each tested configuration. For this test condition, there is no published comparable data reported by the manufacturer.

Despite the dispersion of the results, the data show a lower toughness of the upright samples. This is because the plastic deformation mechanisms that develop in the sample during a high-strain rate (impact) are located at the crack tip of the notch. The crack propagation occurs under a crack opening in mode I, that is, the cross section for where it happens the crack propagation is subjected at tensile stress. Z-axis-printed configurations have the worst impact performance since the interlayer unions on the resistant cross section are perpendicular to the tensile stress. These conclusions run parallel to those reached in the bending tests. The higher impact strength performance is observed for samples with intralayer filaments perpendicular to the opening stress plane. In X-Flat and X-Edge 90° configurations, the better performance compared with the Z-Flat configuration is attributed to the fact that the contour filament dominates the strength section.

### 3.5. Fatigue Mechanical Performance

**Figure 11:**
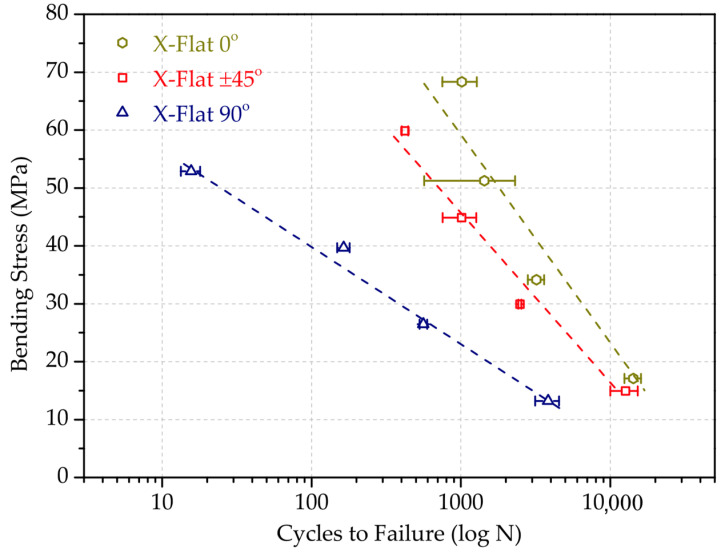
SN curves comparing the effect of the raster angle for X-Flat test configuration.

**Figure 12:**
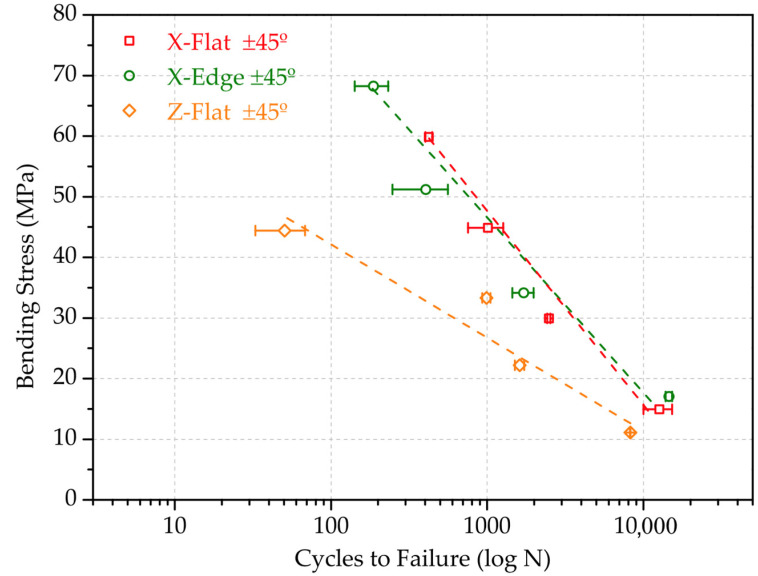
SN curves comparing the effect of the orientation, for samples with ±45° raster angle.

The results of the fatigue tests were analysed based on the SN curves, with respect to the number of cycles to failure, N(S), when a material is repeatedly cycled through a given stress range S. The stress range was defined as 80%, 60%, 40% and 20% percentage of the maximum bending stress determined on Section 3.2. This range was calculated for each sample test configuration. Table 7 outlines the mass and printing time results, together with the cycles to failure results of each sample. Results correspond to the arithmetic mean of three specimens with the same combination of parameters. As representative examples, Figure 11 and Figure 12 depict the SN curves, comparing the effect of the raster angle for X-Flat test configuration, and the SN curves, comparing the effect of the orientation for samples with ±45° raster angle, respectively. This selection is because the X-Flat configuration and ±45° raster angle is commonly used in AM printing strategies.

As shown, overall, the printing configuration has a substantial but unequal influence on fatigue performance. The higher fatigue resistance corresponds to the specimens with the rasters oriented at 0°, coinciding with the direction of the normal stresses. Conversely, the lowest fatigue strength was obtained in the specimens with the rasters at 90°, since samples have intralayer filaments parallel to the stress plane, which favours the crack to form and expand. These conclusions run parallel to those reached in the bending tests. In the case of the X-Edge and Z-Flat configurations (not shown, see Table 7), the differences between the orientations of the rasters are minimal. This is attributed to the fact that the contour filament dominates the strength section.

Regarding the effect of the orientation for samples with the same raster angle, it can be concluded that the Z-axis-printed configurations result in the worst bending fatigue performance due to the separation between their layers, while Flat and Edge setups show similar trends. For 0° (not shown, see Table 7) and 45° rasters, the performance is equivalent. For 90° (not represented), the outstanding fatigue resistance is again due to the contour filament effect. 

## 4. Conclusions

From the obtained results, presented and discussed in previous subsections, the following conclusions were drawn:

- The combination of printing parameters does not have a notable impact on the mass of the specimens. However, in terms of production time, upright samples triple the printing time since these are formed by a much higher number of layers than those printed in the other axes, and because, between layers manufacturing, purging of the tips occurs.
- Overall, results demonstrate the inherent stiffness anisotropy of the FFF technique, but it does not become as prominent as initially expected. This fact is attributed to the quality of the joints between coplanar filaments (intralayer unions) and adjacent layers (interlayer unions) due to the use of a temperature chamber which allows reducing the thermal shock that occurs when the extruded filament is deposited and contacts the previously built layer.
- Significant differences between test orientations are found in the strength data analysis, particularly for Z-printed samples, showing the weakness of the upright printing configuration. This result is because the fracture in the Z-direction tests leads to the separation of two adjacent layers, resulting in a mostly brittle type of failure. Hence, results state the lower resistance of the joints between layers compared with that of the filament polymer itself.
- For the printing configurations studied, the obtained results are generally lower than those reported by the manufacturer, except for the tensile modulus, the average value of which is close to the reference value. The most significant differences are again obtained for the Z specimens. These results demonstrate the importance of analysing the mechanical performance of the specific printing configurations to be used, as the behaviour proves to be highly dependent on multiple AM manufacturing variables.
- Regarding the fatigue strength, the print configuration plays a significant role, influenced both by the raster angle and the print orientation. For low-loading percentages in most configurations, the finite life (10,000 cycles) is reached, except for X-Flat 90° and Z-Flat ± 45°. Therefore, it would be recommended to consider 50% of the ultimate flexural stress reported in the graphs for the designs. The differences are more pronounced for higher load percentages and, therefore, are not recommended unless higher safety factors are considered.
- In terms of overall mechanical performance, among the analysed configurations, it can be concluded that the X-Flat configuration is the one with reliable performance. The difference is most remarkable when manufacturing time is considered.
- The study showed that the obtained results are far from the documented properties of human bone. Specifically, the maximum tensile and shear stiffness of PC-ISO achieved is around 10% of the documented stiffness of cortical bone (tensile modulus 13.48–20.6 GPa and shear modulus 4.52–6.23 [40]). For tensile strengths, the values achieved are closer, between 42% and 77% of the documented strength data (80–150 MPa [41]), but the variability is higher. Nevertheless, the results obtained for PC-ISO provide stiffness moduli close to the trabecular bone (1.78–2.17 GPa [40]).
- Finally, this experimental evidence demonstrates that despite the biocompatibility certification of the PC-ISO material, its use in structural applications may require doping with other materials that contribute to an increase in mechanical performance. The data presented in the paper will contribute to the design of elements with lower structural requirements such as orthoses, shells, and surgical meshes.

## Figures and Tables

**Table 1:** Dimensions of the specimens for each mechanical characterisation test.

Test Specimen Dimensions as Defined in ASTM D638-Type IV (Tensile Testing) [34]	
Minor section width, *W*	6 mm
Major section width, *WO*	19 mm
Length area minor section, *L*	33 mm
Total length, *LO*	115 mm
Calibrated length, *G*	25 mm
Specimen thickness, *T*	4 mm
Minor section width, *W*	6 mm
Major section width, *WO*	19 mm
**Test Specimen Dimensions as Defined in ASTM D790 (Flexural Testing)** [35]	
Length, *L*	127 mm
Depth, *d*	4 mm
Width, *w*	10 mm
**Test Specimen Dimensions as Defined in ASTM D5379 (Shear Testing)** [36]	
Total width, *d_1_*	20 mm
Width in the notch, *w*	12 mm
Depth, *h*	4 mm
Length, *L*	76 mm
Notch angle	90°
Radius at notch angle, *r*	1.3 mm
**Test Specimen Dimensions as Defined in ISO 179-1 (Charpy Impact Testing)** [37]	
Length, *L*	78 mm
Depth, *d*	4 mm
Width, *w*	10 mm
**Test Specimen Dimensions as Defined in ASTM D7774 (Fatigue Testing)** [38]	
Length, *L*	77.2 mm
Depth, *d*	4 mm
Width, *w*	10 mm

**Table 2:** Printing configurations used on each standard mechanical test. All samples were printed with a 0 mm air gap (solid), one contour raster, and normal surface style. See Figure 2 for orientation details.

Configuration	Printing Orientation	Sample Orientation	Raster Angle (°)
1	X	Flat	0
2	X	Flat	±45
3	X	Flat	90
4	X	Edge	0
5	X	Edge	±45
6	X	Edge	90
7	Z	Flat	0
8	Z	Flat	±45
9	Z	Flat	90

**Table 3:** Mass, printing time, and test results of each tensile test configuration.

Configuration		Mass (g)	Printing Time (min)	Tensile Modulus (MPa)	Tensile Strength (MPa)	Strain at Maximum Stress (%)
1	X-Flat 0°	6.62 ± 0.31%	8	2236 ± 6%	62 ± 12%	4.03 ± 15%
2	X-Flat ± 45°	6.86 ± 0.76%	9	2510 ± 7%	65 ± 1%	4.83 ± 3%
3	X-Flat 90°	6.74 ± 0.15%	10	2185 ± 3%	41 ± 5%	3.10 ± 3%
4	X-Edge 0°	5.99 ± 0.35%	8	2110 ± 3%	59 ± 1%	4.99 ± 5%
5	X-Edge ± 45°	6.83 ± 0.39%	12	2114 ± 1%	53 ± 3%	4.28 ± 6%
6	X-Edge 90°	6.64 ± 0.09%	13	2379 ± 2%	62 ± 3%	3.77 ± 1%
7	Z-Flat 0°	6.06 ± 0.91%	44	1976 ± 2%	26 ± 13%	1.36 ± 17%
8	Z-Flat ± 45°	6.80 ± 0.15%	43	1857 ± 1%	28 ± 8%	1.73 ± 10%
9	Z-Flat 90°	6.64 ± 0.40%	40	1806 ± 4%	30 ± 4%	1.56 ± 8%

**Table 4:** Mass, printing time, and test results of each three-point bending test configuration.

Configuration		Mass (g)	Printing Time (min)	Flexural Modulus (MPa)	Bending Strength (MPa)	Strain at Maximum Stress (%)
1	X-Flat 0°	5.48 ± 0.21%	7	1871 ± 1%	85.4 ± 1%	7.1 ± 4%
2	X-Flat ± 45°	5.77 ± 0.10%	9	1654 ± 8%	74.8 ± 4%	7.6 ± 9%
3	X-Flat 90°	5.75 ± 0.17%	10	1674 ± 1%	66.1 ± 2%	5.0 ± 6%
4	X-Edge 0°	5.08 ± 0.30%	8	1793 ± 3%	78.4 ± 1%	6.7 ± 5%
5	X-Edge ± 45°	5.78 ± 0.10%	12	1921 ± 2%	85.3 ± 4%	6.6 ± 5%
6	X-Edge 90°	5.70 ± 0.27%	13	1715 ± 2%	72.7 ± 2%	6.0 ± 3%
7	Z-Flat 0°	5.20 ± 0.80%	44	1759 ± 1%	54.2 ± 5%	2.7 ± 4%
8	Z-Flat ± 45°	5.76 ± 1.20%	43	1715 ± 1%	55.5 ± 6%	3.6 ± 14%
9	Z-Flat 90°	5.55 ± 0.28%	40	1627 ± 3%	44.9 ± 4%	4.4 ± 5%

**Table 5:** Mass, printing time, and test results of each in-plane shear test configuration.

Configuration		Mass (g)	Printing Time (min)	Shear Modulus (MPa)	Shear Strength (MPa)
1	X-Flat 0°	6.71 ± 0.48%	8	546 ± 7%	37.1 ± 11%
2	X-Flat ± 45°	6.77 ± 0.52%	8	586 ± 9%	34.9 ± 3%
3	X-Flat 90°	6.84 ± 0.25%	9	629 ± 8%	32.0 ± 5%
4	X-Edge 0°	5.89 ± 0.35%	12	785 ± 5%	26.5 ± 4%
5	X-Edge ± 45°	6.74 ± 0.09%	15	590 ± 2%	33.6 ± 2%
6	X-Edge 90°	6.57 ± 0.43%	17	670 ± 6%	28.6 ± 4%
7	Z-Flat 0°	5.98 ± 0.79%	26	682 ± 11%	28.2 ± 4%
8	Z-Flat ± 45°	6.77 ± 0.23%	31	634 ± 10%	30.5 ± 7%
9	Z-Flat 90°	6.63 ± 0.38%	33	486 ± 15%	25.9 ± 8%

**Table 6:** Mass, printing time, and test results of each Charpy impact test configuration.

Configuration		Mass (g)	Printing Time (min)	Absorbed Energy (J/cm^2^)
1	X-Flat 0°	3.76 ± 0.23%	5	3.31 ± 17%
2	X-Flat ± 45°	3.79 ± 0.11%	6	2.86 ± 14%
3	X-Flat 90°	3.81 ± 0.21%	7	2.48 ± 10%
4	X-Edge 0°	3.55 ± 0.31%	8	2.95 ± 15%
5	X-Edge ± 45°	3.59 ± 0.24%	9	3.25 ± 24%
6	X-Edge 90°	3.62 ± 0.17%	10	2.68 ± 21%
7	Z-Flat 0°	3.65 ± 0.64%	28	1.22 ± 7%
8	Z-Flat ± 45°	3.64 ± 0.53%	27	1.26 ± 11%
9	Z-Flat 90°	3.65 ± 0.41%	26	1.05 ± 9%

**Table 7:** Mass, printing time, and test results of each fatigue test configuration.

Configuration		Mass (g)	Printing Time (min)	Cycles to Failure at Stress Level			
				80%	60%	40%	20%
1	X-Flat 0°	3.75 ± 0.36%	5	1016 ± 26%	1437 ± 60%	3206 ± 12%	14,245 ± 13%
2	X-Flat ± 45°	3.78 ± 0.14%	6	423 ± 5%	1011 ± 25%	2480 ± 2%	12,623 ± 21%
3	X-Flat 90°	3.77 ± 0.27%	7	16 ± 15%	164 ± 9%	562 ± 6%	3838 ± 18%
4	X-Edge 0°	3.54 ± 0.35%	7	1082 ± 27%	1354 ± 40%	3504 ± 20%	13,589 ± 15%
5	X-Edge ± 45°	3.57 ± 0.30%	9	187 ± 24%	404 ± 39%	1715 ± 16%	14,549 ± 5%
6	X-Edge 90°	3.60 ± 0.31%	10	953 ± 13%	1519 ± 27%	3563 ± 9%	20,009 ± 27%
7	Z-Flat 0°	3.63 ± 0.59%	28	122 ± 27%	432 ± 21%	2647 ± 11%	16,445 ± 6%
8	Z-Flat ± 45°	3.62 ± 0.60%	27	51 ± 35%	989 ± 6%	1614 ± 7%	8236 ± 1%
9	Z-Flat 90°	3.62 ± 0.33%	26	255 ± 6%	959 ± 22%	4075 ± 23%	39,332 ± 2%

## Data Availability

Data are contained within the article.
